# Glutathione Peroxidase 1 Promotes NSCLC Resistance to Cisplatin via ROS-Induced Activation of PI3K/AKT Pathway

**DOI:** 10.1155/2019/7640547

**Published:** 2019-03-27

**Authors:** Baishen Chen, Zhuojian Shen, Duoguang Wu, Xuan Xie, Xia Xu, Liangzhan Lv, Honglue Dai, Ju Chen, Xiangfeng Gan

**Affiliations:** ^1^Department of Thoracic Surgery, Guangdong Provincial Key Laboratory of Malignant Tumor Epigenetics and Gene Regulation, Sun Yat-sen Memorial Hospital, Sun Yat-sen University, Guangzhou 510120, China; ^2^Department of Cardiothoracic Surgery, Renmin Hospital, Hubei University of Medicine, Shiyan 442000, China; ^3^Lung Cancer Research Center of Sun Yat-sen University, Guangzhou 510060, China; ^4^Department of Cardiothoracic Surgery, The Fifth Affiliated Hospital of Sun Yat-sen University, Zhuhai 519000, China

## Abstract

**Purpose:**

Reactive oxygen species (ROS)-induced cytotoxicity is an important mechanism by which cisplatin kills tumor cells. Glutathione peroxidase family (GPXs) is an important member of antioxidant system which metabolizes intracellular ROS and maintains homeostasis of cells. Altered expressions of GPXs enzymes, especially GPX1, have been described in a variety of human cancers. However, their functional roles in cisplatin-based chemoresistance in human malignancies including non-small cell lung cancer have never been explored.

**Methods:**

A panel of NSCLC cell lines were selected for this study. GPX1 expression was detected using quantitative RT-PCR and Western blot. Cisplatin-induced cell killing was analyzed by CCK8 assay. Intracellular ROS levels were detected by fluorescence-based flow cytometry analysis. In vitro overexpression and knockdown of GPX1 expression were performed using GPX1 expression vector and siRNA approaches. Protein levels of PTEN, NF-*κ*B, BCL2, Bax, and phosphorylated AKT were detected with western blot analysis using specific antibodies.

**Results:**

GPX1 expression was upregulated in a subset of NSCLC cell lines resistant to cisplatin treatment. Expression vector-mediated forced overexpression of GPX1 significantly increased cisplatin resistance in NSCLC cell lines, whereas RNA inference-mediated downregulation of GPX1 could restore sensitivity to cisplatin. Overexpression of GPX1 significantly suppressed elevation of intracellular ROS and activation of AKT pathway when NSCLC cell lines were exposed to different concentrations of cisplatin. Activation of the AKT pathway inhibited proapoptotic cascade and subsequently led to cisplatin resistance in NSCLC cells. Inhibition of NF-*κ*B by its chemical inhibitor BAY can significantly downregulate GPX1 expression and restore the cisplatin sensitivity of the cell lines resistant to cisplatin.

**Conclusions:**

Our findings suggested that overexpression of GPX1 is a novel molecular mechanism for cisplatin-based chemoresistance in NSCLC. GPX1 overexpression blocks cisplatin-induced ROS intracellular accumulation, activates PI3K-AKT pathway by increased AKT phosphorylation, and further leads to cisplatin resistance in NSCLC cells. Inhibition of NF-*κ*B signaling may be an alternative approach for restoring cisplatin sensitivity for NSCLC cells resistant to cisplatin-based chemotherapy.

## 1. Introduction

Lung cancer is the leading cause of cancer-related deaths worldwide [1], and non-small cell lung cancer (NSCLC) accounts for approximately 85% of overall lung cancer incidence [1]. Cisplatin (DDP)-based multidrug combination chemotherapy is the standard chemotherapy treatment currently used for NSCLC patients [2, 3]. However, emergence of resistance to the drugs, particularly DDP, is one the most common causes leading to chemotherapy failure in NSCLC patients [3]. Therefore, further understanding of the mechanisms of chemoresistance is essential for developing novel therapeutic strategies and improving clinical outcomes of chemotherapy for NSCLC patients.

DDP induces DNA intrastrand crosslinks in tumor cells and subsequently initiates apoptotic programs via causing DNA damage. Chemoresistance to DDP is a complex phenotype that involves multiple cellular processes, including alterations in drug influx or efflux, apoptosis, cell cycle, DNA repair capacity, and other mechanisms required for DNA damage repair [3]. Reactive oxygen species (ROS) is an important biological product of normal cellular metabolism and accumulating evidence suggests that ROS functions as an essential regulator for many biological processes including survival, proliferation, and apoptosis of cells. Apoptosis initiated via intracellular accumulation of ROS has been well accepted as a fundamental mechanism contributing to antitumor mechanism of DDP [4, 5].

ROS is generated in mitochondria when cells are exposed to environmental stress including cisplatin challenge [6, 7]. Excessive accumulation of ROS causes DNA damage and subsequently activates apoptotic machinery for cell death. Antioxidant enzymes are an enzyme family which eliminates excessive ROS and maintains genomic integrity and hemostasis of cells. In antioxidant enzyme family, glutathione peroxidase, especially GPX1, is the widely expressed and most important member metabolizing ROS. Several previous studies have reported that expressions of GPX enzymes were altered in various cancer types: upregulation: lung cancer [8], breast cancer [9]; downregulation: colorectal cancer [10], thyroid cancer [11], gastric cancer [12]. However, the effects of altered GPX enzyme expression on various malignant phenotype including chemoresistance to cisplatin treatment have never been described [4, 5, 13]. No previous study has reported the alteration and functional implication of GPX enzyme in non-small cell lung cancer so far.

Our previous study found that promoter of GPX3 gene was hypermethylated in head and neck cancer, and GPX3 hypermethylation is associated with cisplatin-based chemoresistance and a poor prognosis in head and neck cancer [14]. This finding suggests that GPX enzymes may paly critical functional roles in development of chemoresistance to cisplatin-based chemotherapy in human cancers. The finding leads us to examine the roles of GPX1, a widely and abundantly expressed member in GPX family, in cancer chemoresistance. By screening expression of GPX1 in a panel of NSCLC cell lines, we found that GPX1 is overexpressed in a subset of cell lines resistant to cisplatin treatment. In this study, we performed functional characterization of GPX1 in chemoresistance to cisplatin-based chemotherapy in NSCLC.

The PI3K-AKT pathway is a vital signal pathway essential for proliferation, differentiation, apoptosis, and drug resistance of cancer cells [15]. Aberrant activation of PI3K/AKT pathway is frequently observed in cancer cells resistant to cisplatin treatment [16–18]. Interplay between antioxidant redox system and PI3K/AKT pathway in cancer chemoresistance currently remains unclear.

## 2. Materials and Methods

### 2.1. Cell Lines and Cell Culture

A total of 4 NSCLC cell lines (A549, H1975, H460, and H1650) were selected for this study (including GLC-82, H1993, H2170, Spc-a1, and H1299): adenocarcinoma lung cancer: A549, GLC-82, H1975, H1993, Spc-a-1, H1650, H1299; large cell lung cancer: H460; squamous carcinoma: H2170. All cell lines were obtained from the cell line bank in the Sun Yat-sen University (Guangzhou, China). The cells were cultured in RPMI1640 medium (Gibco, Gaithersburg, MD, USA) containing 10% fetal bovine serum (FBS) at 37°C in a humidified atmosphere containing 5% CO_2_.

### 2.2. GPX1 Expression Vector and siRNA

A small interfering RNA (siRNA) specifically targeting GPX1 and a scrambled control siRNA were constructed by Invitrogen (Carlsbad, California, US). The sequences of the GPX1 siRNA were as follows: sense, GGUACUACUUAUCGAGAAUTT; antisense, AUUCUCGAUAAGUAGUACCTT. The sequences of scrambled control siRNA were as follows: sense, UUCUCCGAACGUGUCACGUTT; antisense, ACGUGACACGUUCGGAGAATT. GPX1 expression vector was constructed using a pcDNA 3.1 expression vector (Invitrogen, Carlsbad, California, US). Full-length GPX1 coding sequences were PCR amplified and cloned into a pcDNA 3.1 expression vector according to a standard procedure. Sanger DNA sequencing was used to verify the expression constructs. For all in vitro experiments of exogenous GPX1 expression and siRNA transfection, Lipofectamine 2000 Transfection Reagent (Invitrogen, Carlsbad, California, US) was used as a transfection agent according to the standard protocol suggested from the manufacturer. After forty-eight-hour transfection, the transfection efficiency of the GPX1 overexpression vector and the siRNA was verified via RT-PCR.

### 2.3. CCK-8 Assay

Cell proliferation was assessed using CCK-8 assay. Cells were seeded into 96-well plates at 5000/well for culture. After 48 hours, the optical density (OD value) was measured by the Cell Counting Kit-8 (CCK-8, Dojindo, Japan) assay. CCK-8 solution (10 *μ*L) and 90 *μ*L medium were added to each well, and the plates were incubated at 37°C for 1 h. Then, the OD value was measured at 450 nm with a Microplate Spectrophotometer (Thermo Scientific, Belmont, Massachusetts, US).

To test the cisplatin sensitivity of cells, cells (3 × 10^3^) were seeded in 96-well plates. After overnight culture, the cells were treated with various concentrations (0, 2.5, 5, 10, 20, and 40 *μ*mol/L) of cisplatin (Sigma, Santa Clara, California, US) and a CCK-8 assay was performed to examine the cytotoxicity of cisplatin after 72 hours of treatment. The relative survival of the cells (%) was determined by optical density (OD) value.

### 2.4. EGF, LY294002, or BAY 11-7082 Treatment of NSCLC Cells

Cells were treated with 25 *μ*M LY294002 (Calbiochem, La Jolla, CA) for 2 h or stimulated with EGF (40ng/ml) (Sigma) for 30 min after 48 h transfection by siGPX1(A549)/GPX1 vector(H1640). NF-*κ*B inhibitor BAY 11-7082 was used for assessment of the effect of NF-*κ*B on cisplatin responses of lung cancer cell lines. A549 and H1975 cells were inoculated at 1 × 10^6^ cells/well in 6-well plates. After overnight culture, these cells were treated with 10 nmol/L of BAY 11-7082 (BAY) (Beyotime, Shanghai, China) for 1 hour and washed with phosphate-buffered saline. Treated cells were collected and used for further molecular and functional analysis.

### 2.5. Reverse Transcription Polymerase Chain Reaction (RT-PCR) Analysis

RNA extraction and purification from cell lines were performed using TRIzol™ reagent (Sigma, Santa Clara, California, US) according to a standard protocol. Two micrograms of total RNA were reverse-transcribed into cDNA using the Prime Script RT reagent kit (TaKaRa, Otsu, Japan). Expression level of GPX1 transcript was assessed by quantitatively amplifying the synthesized cDNA using a CFX96 Real-Time PCR Detection System (Bio-Rad, Hercules, California, US) by an SYBR Green method (TaKaRa, Otsu, Japan). The primers were purchased from TaKaRa (Otsu, Japan), and GAPDH was used as an endogenous control. The primer sequences were listed as follows: GAPDH, 5′-GCACCGTCAAGGCTGAGAAC-3′ (sense) and 5′-TGGTGAAGACGCCAGTGGA-3′ (antisense); GPX1, 5′-GCGGGGCAAGGTACTACTTA-3′ (sense) and 5′-CTCTTCGTTCTTGGCGTTCT-3′ (antisense). Real-time amplification was performed according to the protocol suggested by the manufacturer (TaKaRa, Otsu, Japan).

### 2.6. Western Blot Analysis

Protein expressions of GPX1, PTEN, PDK-1, AKT, Bcl-2, Bax, and Nf-*κ*B were detected by western blot using specific primary antibodies (Gene Tex, Irvine, California, US). Antibody against GAPDH (Kangcheng Biology, Shanghai, China) was used as a loading normalization control. After BAY 11-7082 treatment or transfection experiments, the cells were collected and the total protein was extracted from the cells. Protein (30 *μ*g) was loaded and separated using a 10% sodium dodecyl sulfate polyacrylamide gel electrophoresis (SDS-PAGE) (Beyotime, Shanghai, China) gel and transferred to an Immobilon-P Transfer Membrane (PVDF) (Beyotime, Shanghai, China). The membranes were blocked with 5% nonfat milk in Tris-buffered saline (TBS) containing 0.1% Tween-20 for 1 h at room temperature. The blots were probed with the selected primary antibodies overnight at 4°C, washed in TBST, and further incubated with a species-specific horseradish peroxidase-conjugated secondary antibody (anti-rabbit, ExCell Bio, Shanghai, China) for 1 hour at room temperature. The western blot process was performed according to standard protocols. An enhanced chemiluminescence detection method (Pierce ECL Western Blotting Substrate) (Thermo Scientific, Belmont, Massachusetts, US) was used to visualize the blots.

### 2.7. Detection of Intracellular ROS

Intracellular ROS was detected using an oxidation-sensitive fluorescent probe (DCFH-DA) (Beyotime, Shanghai, China). After transfection with siRNA and expression vector or treatment with BAY 11-7082, cells were plated in 6-well plates and then treated with 2.5 *μ*M or 5 *μ*M cisplatin for 72 hours at 37°C. The cells were collected and washed twice in phosphate-buffered saline (PBS). The cells were then incubated with 10 *μ*mol/L DCFH-DA at 37°C for 20 min according to the manufacturer's instructions. Then, fluorescence was detected by a FACSCalibur flow cytometer (BD Biosciences, MA) [19, 20].

### 2.8. Statistical Analysis

All data are presented as the mean ± standard deviation (SD). The data were analyzed with Student's t-test, and p-values <0.05 were considered significant. All statistical analyses were performed with the SPSS program package 13.0 (SPSS Inc., Chicago, IL, USA).

## 3. Results

### 3.1. Overexpression of GPX1 Promoted Cisplatin Resistance in NSCLC Cells

**Figure 1:**
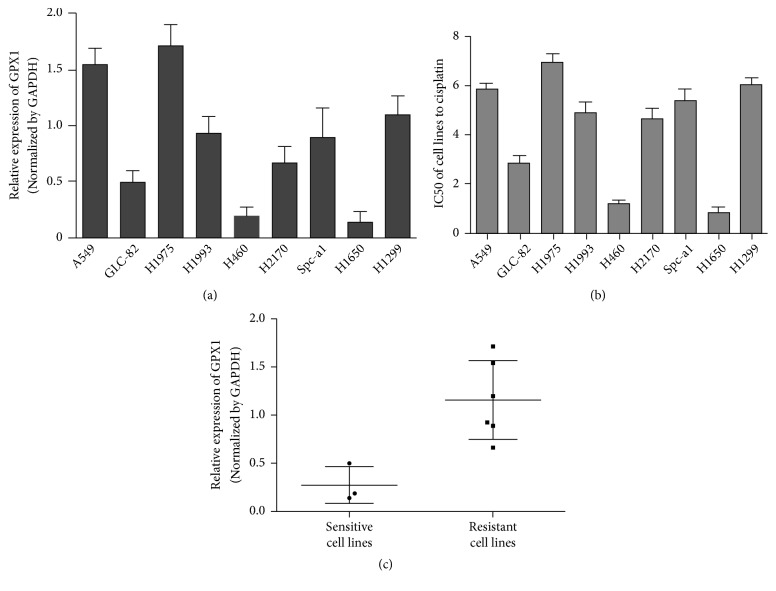
*The relationship of the expression of GPX1 and IC50 in NSCLC cell lines.* (a) The relative expression of GPX1 in 9 NSCLC cell lines was detected by quantitative RT-PCR. (b) The IC50 of 9 NSCLC cell lines to cisplatin. (c) The relationship of the expression of GPX1 and IC50 in NSCLC cell lines.

Relative expression levels of GPX1 in 9 NSCLC cell lines were detected by quantitative real-time* RT-*PCR (Figure 1(a)) using GAPDH as an endogenous normalization control. Cisplatin sensitivity (IC50) of the 9 NSCLC cell lines was determined using in vitro cytotoxicity assay (CCK-8 assay, Figure 1(b)). Based on the cut-off IC50 cisplatin concentration of 4 *μ*M, the cell lines were classified into cisplatin sensitive (IC50 ≤ 4 *μ*M) and resistant (> 4 *μ*M) groups. A relationship of GPX1 expression and cisplatin sensitivity was found between cisplatin sensitive (GLC-82, H460, and H1650) and resistant cell lines (A549, H1975, H1993, H2170, Spc-a-1, and H1299). As shown in Figure 1(c), the mean GPX1 expression level in 6 resistant cell lines was significantly higher than that in 3 sensitive cell lines, suggesting that GPX1 might be involved in cisplatin resistance of lung cancer.

**Figure 2:**
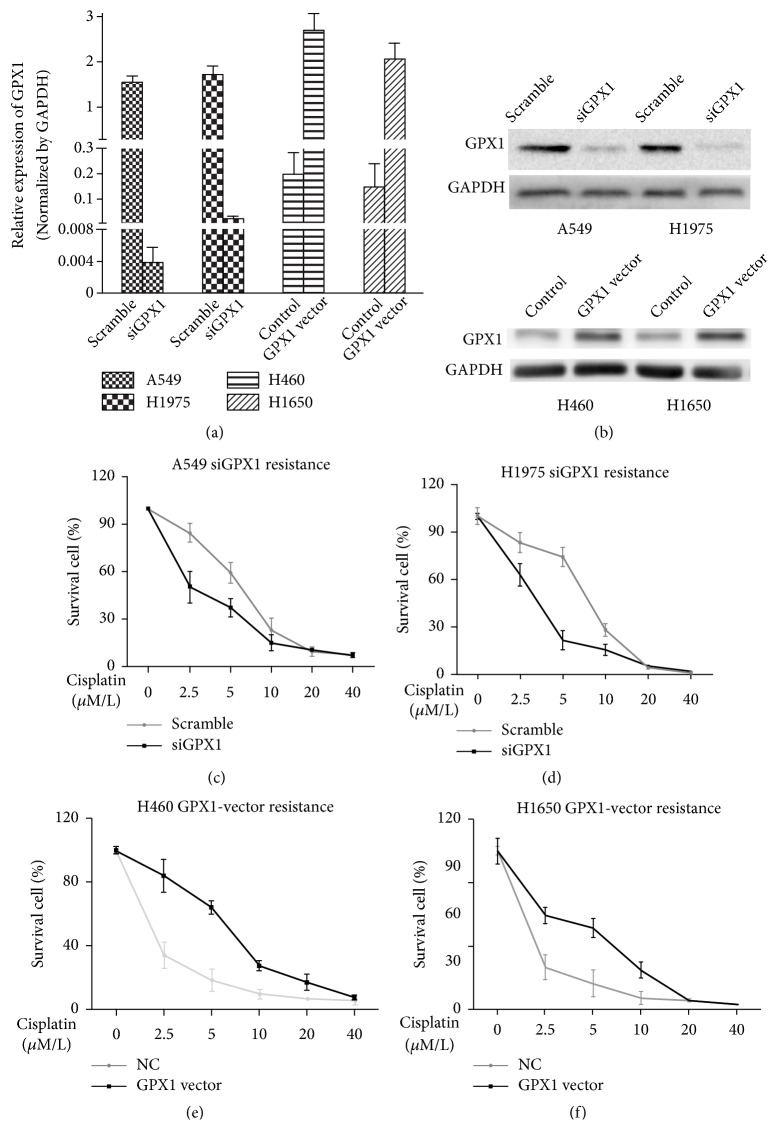
*Increased expression of GPX1 promotes cisplatin resistance in NSCLC cells*. ((a), (b)) To confirm the transfection efficiency, we used quantitative RT-PCR (a) and western blotting (b). Decreasing ((c) and (d)) or increasing ((e) and (f)) GPX1 expression influenced the cisplatin resistance in NSCLC cells. Reduced GPX1 expression downregulates cisplatin resistance, and increased GPX1 expression enhances cisplatin resistance (^*∗*^P<0.05). Highly significant effects were observed at cisplatin concentrations of 2.5 and 5 *μ*mol/L.

To determine the potential functional role of GPX1 in cisplatin resistance of lung cancer cells, two cell lines (A549 and H1975) with highest GPX1 expression and two (H460 and H1650) with relatively low GPX1 expression were selected for further in vitro functional study. We exogenously overexpressed GPX1 expression in the two cell lines with low GPX1 expression (H460 and H1650) by transfection of GPX1 expression vector and suppressed endogenous GPX1 expression in cell lines with high GPX1 expression (A549 and H1975) using transfection of GPX1 siRNA (Figures 2(a) and 2(b)). Forty-eight hours after transfections, the transfected cell lines were cultured in 96-well plates for 72 hours with different concentrations of cisplatin (0, 2.5, 5, 10, 20, and 40 *μ*mol/L). As shown in Figures 2(c) and 2(d), downregulation of GPX1 in GPX1 high expression and cisplatin resistant cell lines A549 and H1975 dramatically enhanced their sensitivity to cisplatin treatment (IC_50_: A549, 5.876_(Scrambled)_ vs. 2.666_(siGPX1)_; H1975, 6.946_(Scrambled)_ vs. 3.007_(siGPX1)_). In contrast, exogenous overexpression of GPX1 in GPX1 low expression and cisplatin sensitive cell lines H460 and H1650 significantly reduced their sensitivity to cisplatin challenge (H460, 1.230_(NC)_ vs. 6.643_(GPX1 vector)_; H1650 0.849_(NC)_ vs. 4.090_(GPX1 vector)_) (Figures 2(e) and 2(f)).

### 3.2. Overexpression of GPX1 Induces DDP-Resistance by Blocking Accumulation of Intracellular ROS

ROS has been well demonstrated to be an important mediator for cisplatin-induced cell killing. Since GPX1 plays a fundamental role in metabolizing intracellular ROS and maintaining hemostasis of cells, it is reasonable to hypothesize that elimination of ROS induced by cisplatin treatment may be the associated mechanism for GPX1 induced resistance to cisplatin in lung cancer.

**Figure 3:**
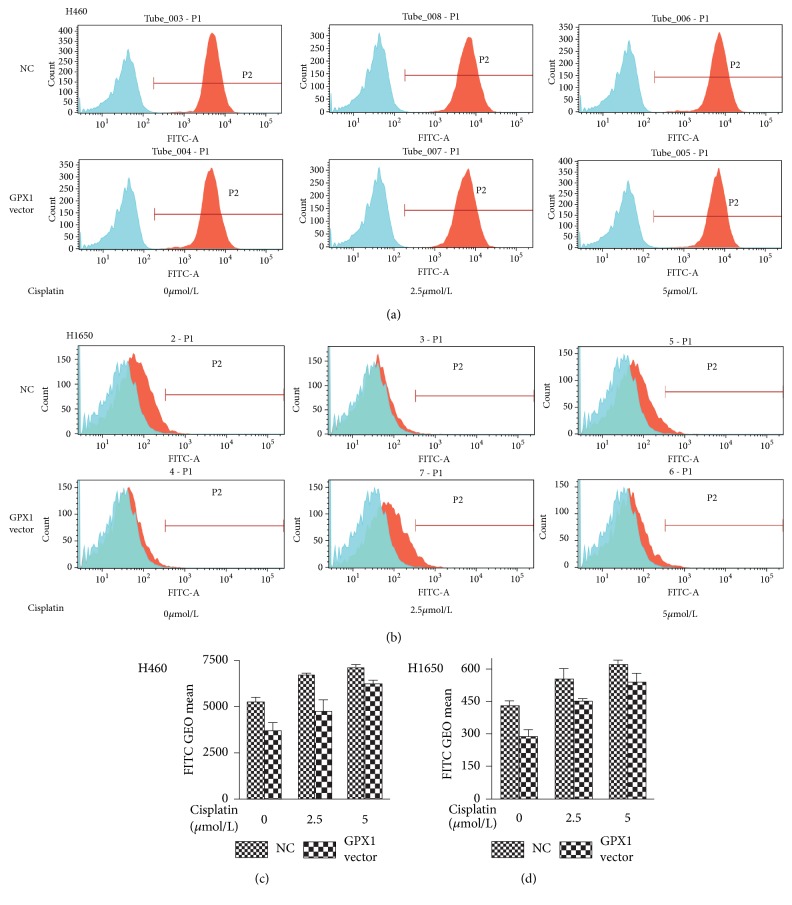
*Increased GPX1 expression reduced intracellular ROS accumulation in NSCLC cells.* The H460 (a) and H1650 (b) were transfected with GPX1 expression vector, and GEO mean value was detected by a flow cytometer after cells were treated with different concentrations of cisplatin for 72 h in culture. Along with the increased concentrations of cisplatin, intracellular ROS (GEO mean) in GPX1 vector transfected cells decreased as compared to empty vector transfected control cells ((c) and (d)). The difference of intracellular ROS levels between GPX1 vector and empty vector transfected cells is statistically significant (P<0.05).

To test our hypothesis above and to determine the role of ROS in GPX1 associated resistance to cisplatin in lung cancer, we compared the dynamic changes of intracellular ROS levels with and without exogenous overexpression of GPX1 gene in cisplatin sensitive cell lines H460 and H1650. After transfection with the GPX1 vector, H460 and H1650 were exposed to treatment with cisplatin concentrations of 2.5 and 5 *μ*M, respectively, and further cultured for additional 72 h. Treated cells were harvested and intracellular ROS was detected by a flow cytometer with fluorescent probe (DCFH-DA). As the finding shown in Figures 3(a), 3(b), 3(c), and 3(d), significantly lower intracellular ROS level was detected in GPX1 vector transfected cells than that in NC (empty vector control) cells. When H460 and H1650 cells were exposed to cisplatin challenge, increasing levels of ROS were detected in both GPX1 vector transfected cells and NC group (Figures 3(a), 3(b), 3(c), and 3(d)). However, intracellular ROS levels remained relatively lower in GPX1 vector transfected H460 and H1650 cells as compared to NC transfected cells upon cisplatin treatment (Figures 3(c) and 3(d)). These findings implicate that GPX1 can functionally diminish intracellular ROS level induced by cisplatin treatment in lung cancer cells.

### 3.3. GPX1 Could Affect DDP-Resistance via PI3K-AKT Pathway

The PI3K-AKT pathway plays an important role in cell survival through activation of antiapoptotic factors and the inhibition of proapoptotic factors [16]. The activation of AKT was initiated by translocation to the plasma membrane immediately after cells are stimulated by various cell survival regulators [21]. AKT becomes fully activated when the protein is phosphorylated by PDK1 and PDK2 at Thr308 and Ser473. PTEN phosphatase was a signaling inhibitor of the PI3K-AKT pathway and could negatively regulate the function of AKT [16, 21]. It has been reported that reduction in PTEN expression that indirectly stimulated PI3K-AKT activity and also reduced intracellular level of ROS, could promote cell survival through activation of AKT pathway [21].

**Figure 4:**
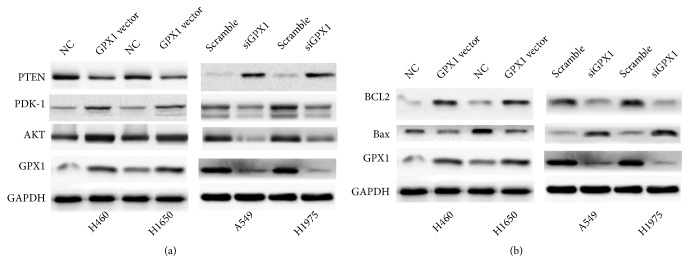
*GPX1 could affect the PI3K-AKT pathway in NSCLC*. (a) In cisplatin sensitive cell lines H460 and H1650, forced expression of GPX1 increased the level of activated AKT (detected using antibody against phosphorylated AKT) whereas downregulation of GPX1 by siRNA reduced the level of activated AKT. (b) Overexpression of GPX1 increased BCL2 level and reduced Bax expression, whereas downregulation of GPX1 inhibited BCL2 expression and increased Bax expression.

To determine the association of the PI3K-AKT pathway with mechanisms in GPX1-induced DDP chemoresistance in NSCLC, we examined the association of expression and activation of PTEN, PDK-1, and AKT with GPX1 expression in NSCLC cell lines. In the experiments stated above (Figures 3(a), 3(b), 3(c), and 3(d)), we demonstrated that the accumulation of ROS was blocked after overexpression of GPX1 in H460 and H1650. In the experiment, we further analyzed the expressions and activation of PTEN, PDK-1, and AKT phosphorylation by western blot in H460 and H1650 cell lines with in vitro forced overexpression and siRNA-induced knockdown of GPX1. As shown in Figure 4(a), in vitro forced overexpression of GPX1 downregulated the protein expressions of PTEN and correspondingly increased PDK1 protein expression and AKT phosphorylation. The finding was further confirmed by siRNA-induced knockdown of GPX1 expression in cell lines A549 and H1975 with high endogenous expression of GPX1 (Figure 4(a)).

BCL2 and Bax are antiapoptotic and proapoptotic factors and are important downstream targets for PI3K-AKT-mediated cell survival. To demonstrate the functional activation of cell survival signaling in NSCLC cells with overexpression of GPX1, expression levels of BLC2 and Bax were analyzed by western blot in cell lines (H460 and H1650) with forced overexpression of GPX1 and in cell lines (A549 and H1975) with siRNA-knockdown expression of GPX1. BCL2 was upregulated in H460 and H1650 with GPX1 forced expression as compared with the empty vector transfected cells while proapoptotic molecule Bax was downregulated in these two cell line models (Figure 4(b)). In contrast, siRNA-induced knockdown of expression of GPX1 suppressed BCL2 expression and upregulated Bax expression in A549 and H1975 cells as compared to scrambled RNA controls (Figure 4(b)). Thus, the above data describes that GPX1 could affect the activity of AKT pathway.

**Figure 5:**
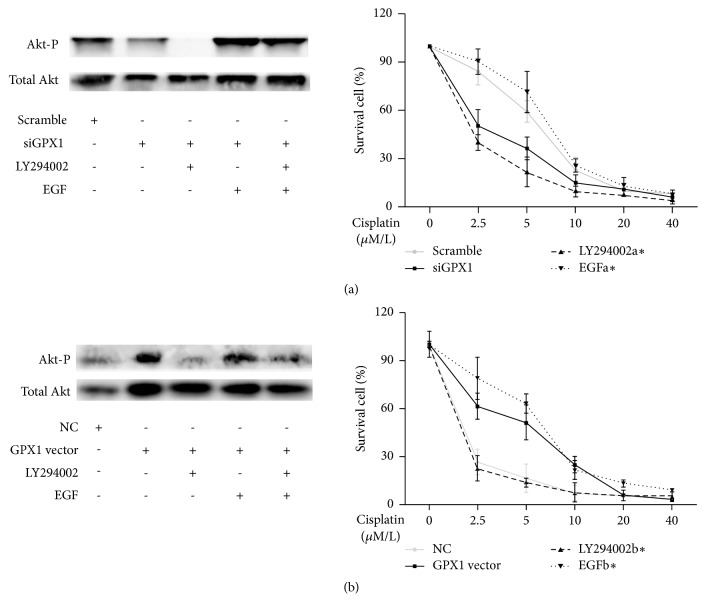
*GPX1 could mediate cisplatin resistance in NSCLC via the PI3K-AKT pathway.* (a) Treatment of GPX1 downregulated A549 cells with EGF, a PI(3)K activator, increased their sensitivity to cisplatin compared to A549 cells treated with siGPX1 alone. Western blot analysis demonstrating an inhibition of AKT phosphorylation in response to LY294002 in GPX1-downregulated A549 cells and regaining the expression of that by treatment of EGF. (b) Treatment of GPX1 upregulated H1650 cells with LY294002, a PI(3)K inhibitor, decreased their sensitivity to cisplatin compared to H1650 cells treated with GPX1 vector alone. (*∗*: LY294002a, LY294002 with siGPX1; EGFa, EGF with siGPX1; LY294002b, LY294002 with GPX1 vector; EGFb, EGF with GPX1 vector.)

To determine the role of GPX1 in the regulation of NSCLC resistance to cisplatin via the AKT pathway, GPX1-downregulated A549 cells were treated with EGF, a PI(3)K activator (Figure 5(a)). Pretreatment with siGPX1 transfection followed by a 30 min treatment with 40 ng/ml EGF regained the sensitivity to cisplatin: IC50: scramble, 5.865 *μ*M (95%CI: 5.226 to 6.581); siGPX1, 2.651 *μ*M (95%CI: 2.095 to 3.354); LY294002 with siGPX1, 1.740 *μ*M (95%CI: 1.344 to 2.254); EGF with siGPX1, 7.021 *μ*M (95%CI: 6.141 to 8.027). Importantly, it is seen as a phenocopying of the response in GPX1-upregulated H1650 cells (Figure 5(b)): IC50: NC, 0.839 *μ*M (95%CI: 0.486 to 1.448); GPX1 vector, 4.223 *μ*M (95%CI: 3.652 to 4.882); LY294002 with GPX1 vector, 0.449 *μ*M (95%CI: 0.190 to 1.062); EGF with GPX1 vector, 5.978 *μ*M (95%CI: 5.126 to 6.972). This suggests that GPX1 could affect DDP-resistance via PI3K-AKT pathway.

### 3.4. BAY 11-7082, a NF-*κ*B Inhibitor, Effectively Downregulated GPX1 and Restored Sensitivity of NSCLC to Cisplatin

Previous studies have demonstrated that NF-*κ*B could regulate GPX1 expression by direct binding to the GPX1 promoter [13]. In addition, activation of NF-*κ*B pathway has been frequently observed in cancer cells resistant to chemotherapy [13]. To elucidate the upstream signaling regulating GPX1 expression in NSCLC cells, we proceeded to examine the role of NF-*κ*B in regulation of GPX1 expression in NSCLC cell lines (A549 and H1975) with elevated expression of GPX1 as well as in development of cisplatin-based chemoresistance of NSCLC.

**Figure 6:**
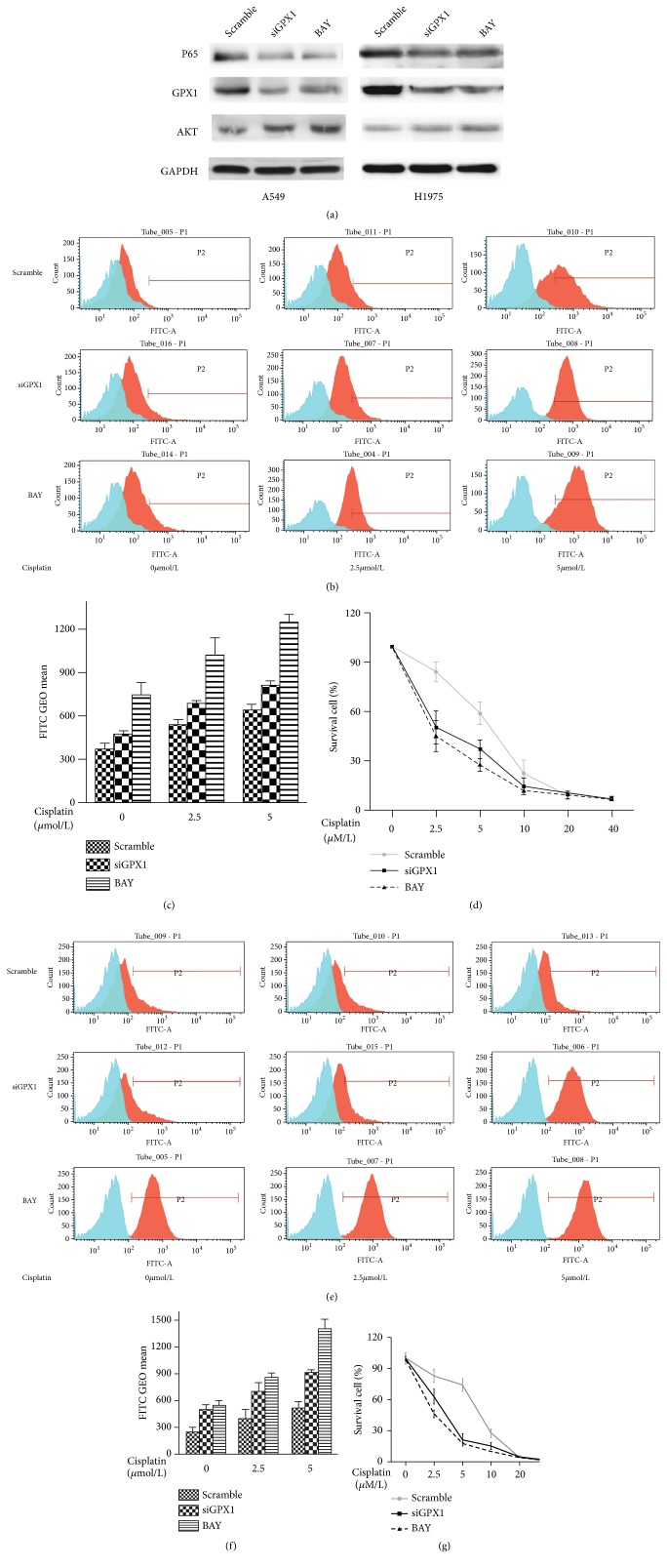
*BAY 11-7082, a NF-κB inhibitor, downregulated intracellular ROS and reduced cisplatin resistance.* (a) A549 and H1975 NSCLC cells treated with BAY 11-7082 showed downregulation of GPX1 expression, and correspondingly AKT phosphorylation was inhibited. The effects of BAY 11-7082 were similar to the effects observed in the same cell lines treated with GPX1 siRNA. ((b), (c), and (d)) In H1975 cells, suppression of GPX1 expression by both siGPX1 and BAY 11-7082 enhanced intracellular ROS accumulation and subsequently restored the sensitivity of the treated cells to cisplatin. ((e), (f), and (g)) A549 cells showed the same findings as H1975 cells when the cells were treated with siGPX1 and BAY 11-7082.

A549 and H1975 with overexpression of GPX1 were treated with BAY 11-7082, a functional inhibitor of NF-*κ*B activity, for 1 hour, and then GPX1 expression and activation of PI3K-AKT pathway of the treated cells were examined. Treatment by NF-*κ*B inhibitor BAY 11-7082 effectively reduced the level of activated form p65 of NF-*κ*B in both A549 and H1975 cells (Figure 6(a)). Analysis of GPX1 and AKT expression in BAY 11-7082 treated A549 and H1975 cells showed that inhibition of NF-*κ*B activity significantly downregulated GPX1 and phosphorylated AKT levels in treated cells (Figure 6(a)). The inhibitory effects of BAY11-7082 on GPX1 and phosphorylated AKT level were similar to the effects of GPX1 siRNA (Figure 6(a)). These finding were consistent with the observations reported in esophageal cancer in a previous study [13]. Tests of effects of BAY 11-7082 on cisplatin response and intracellular ROS level on A549 and H1975 cells further showed that inhibition of NF-*κ*B activity remarkably increased intracellular ROS accumulation and cisplatin sensitivity of the treated cells (Figures 6(b), 6(c), 6(e), and 6(f)). As shown in Figures 6(c) and 6(d), cisplatin IC50 (IC50_cisplatin_) of BAY 11-7082 treated A549 and H1975 cells was 2.066 uM and 2.225 uM as compared to 5.876 uM and 6.946 uM for the untreated A549 and H1975 cells. The effect of NF-*κ*B inhibition on intracellular ROS accumulation and cisplatin sensitivity was similar to that of GPX1 siRNA in both A549 and H1975 cells (Figures 6(b), 6(c), 6(d), 6(e), 6(f), and 6(g)).

## 4. Discussion

Cisplatin is the first drug approved for clinical use for cancer chemotherapy [22]. It has become evident that cisplatin-based chemotherapy could improve the prognosis of patients and prolong survival of patients with various cancer types including non-small cell lung cancer [23, 24]. Currently, cisplatin-based chemotherapy is an accepted standard of care for systemic therapy for patients at advanced stages of NSCLC [23, 24]. However, development of acquired resistance, particularly in colon, lung, and prostate cancers, has been a major challenge in clinical application of cisplatin-based chemotherapy [3, 25]. Despite intensive studies in the past few decades, mechanisms behind development of resistance to cisplatin therapy in NSCLC cancer cells remain unclear.

ROS is formed as byproducts of the normal metabolism of oxygen and plays important roles in cell signaling and homeostasis [26]. When cells are exposed to environmental stress and DNA damage such as cisplatin challenge, intracellular ROS production dramatically increases and triggers apoptotic machinery of cells [26]. Although mechanism of cisplatin cytotoxicity is unclear, induction of excess accumulation of ROS has been proposed to be an essential mechanism for cisplatin-induced killing of cancer cells [22]. Many previous studies from a variety of experimental models observed the correlations of intracellular ROS level and cytotoxic effects of various cytotoxic agents [26, 27]. By analyzing dynamic changes of intracellular ROS, we study the roles of GPX1, an important antioxidant enzyme, in the development of resistance to cisplatin-based therapy in NSCLC.

The fundamental roles of GPXs and glutathione in regulating intracellular ROS level and maintaining homeostasis of intracellular microenvironment have been well demonstrated, and altered expression of GPX family members including GPX1 has been reported in various diseases including cancers [26, 28, 29] (upregulation: lung cancer [8], breast cancer [9]; downregulation: colorectal cancer [10], thyroid cancer [11], gastric cancer [12]). However, association of altered expression of GPX1 with cancer chemoresistance has never been reported previously. In NSCLC, GPX1 and its role in cisplatin-based chemoresistance have not been studied [3, 25]. Our current study found that GPX1 is upregulated in NSCLC cells and increased expression of GPX1 transcript could block cisplatin-induced intracellular ROS accumulation, seemly, activate PI3K-AKT pathway, and further prevent apoptosis of NSCLC cells upon cisplatin challenge. In our knowledge, these findings for the first time demonstrate the association of the altered expression of GPX1 and chemoresistance to cisplatin therapy in NSCLC.

PI3K-AKT pathway is an essential biological mechanism in inhibiting apoptotic processes and promoting cellular survival [21]. Activation of PI3K-AKT pathway by phosphorylating AKT has been implicated as tumorigenesis and progression of many cancer types [30]. Association of functional status of PI3K-AKT pathway with intracellular level of ROS has been previously reported in various experimental models [31, 32]. In HUVEC cell models pretreated with H_2_O_2_, suppression of AKT phosphorylation was observed while intracellular ROS was elevated. Similar results were found in normal neurocytes, VSMCs, and cancer cells [28, 29, 31–34].

Also in Taylor's article [30], it was observed that, in the GPX1-/- murine embryonic fibroblasts (MEFs), the cell viability decreases much more than the wildtype ones when treated with H2O2, because of downregulation of AKT pathway activity. And it was speculated that the lack of a viable PI(3)K pathway may be partially responsible for the increased susceptibility to H2O2-induced cell death seen in the Gpx1-/- cells [30]. In this study, we showed that downregulation of GPX1 in cisplatin resistant NSCLC cell lines A549 and H1975 significantly increased intracellular accumulation of ROS, inhibited AKT phosphorylation, and further promoted proapoptotic activity of the cells upon cisplatin treatment. In contrast, forced overexpression of GPX1 in cisplatin sensitive cell lines H460 and H1650 dramatically abolished intracellular ROS accumulation, enhanced AKT phosphorylation, and suppressed proapoptotic activity of NSCLC cells which were challenged with cisplatin. These results implicate that activation of PI3K-AKT pathway by eliminating intracellular ROS accumulation is one of the critical mechanisms for cisplatin-based chemoresistance in NSCLC cells with GPX1 overexpression.

Upregulation of GPX1 has been reported in several major cancer types, such as lung and breast [8, 9]. The fundamental role of GPX1 in regulating redox system and downstream signaling suggests that altered expression of GPX1 may be an important molecular event in tumorigenesis and progression of human malignancies. However, molecular mechanisms regulating GPX1 expression in cancers have never been demonstrated. A previous study showed that GPX1 promoter contains NF-*κ*B transcription consensus binding sequences and NF-*κ*B can bind to GPX1 promoter and regulate GPX1 expression when cells were exposed to oxidative stress [30]. Moreover, activation of NF-*κ*B pathway has been well demonstrated in development of chemoresistance including cisplatin-based chemotherapy in human cancer. To determine if activated NF-*κ*B is the major molecular event for GPX1 overexpression in NSCLC, we analyzed the effects of NF-*κ*B inhibitor BAY 11-7082 on GPX1 expression, intracellular ROS level, AKT phosphorylation, and cisplatin response in NSCLC cell lines. We showed that treatment with NF-*κ*B inhibitor BAY 11-7082 dramatically downregulated GPX1 expression, promoted AKT phosphorylation activation, increased intracellular ROS production when cells were challenged with cisplatin, and enhanced cisplatin sensitivity in A549 and H1975 NSCLC cells. The effects of BAY 11-7082 treatment on A549 and H1975 cells were similar to those findings observed using siRNA-mediated downregulation of GPX1 expression. The finding implicates that inhibition of NF-*κ*B signaling may be a potential approach for restoring cisplatin sensitivity of NSCLC cells with overexpression of GPX1.

However, as shown in article [30], the PI(3)K-AKT and NF*κ*B pathways act independently of each other. For the limitation of our study, we could not get similar results. It even seemed that AKT pathway activation may be positively related to p65, and further research is needed in future.

Our study demonstrates the association of GPX1-ROS - AKT signaling cascade with cisplatin resistance in NSCLC cells. The findings provide an important basis for understanding the molecular mechanisms for NSCLC cisplatin-based chemoresistance and for developing novel clinical strategies for clinical management of NSCLC patients resistant to cisplatin-based chemotherapy.

## Data Availability

All data included in this study are available upon request by contact with the corresponding author.

## Additional Points

*(i) Significant Findings of the Study*. In this work, we showed that GPX1 might be an important biotarget for cisplatin-based therapy in NSCLC.* (ii) What This Study Adds*. We highlight the crosstalk between GPX1-ROS - AKT cascade bioactivities and cisplatin resistance in NSCLC cells, specifically targeting cisplatin resistance due to aberrant expression of GPX1.
